# Combination of lymphocyte count and albumin concentration as a new prognostic biomarker for rectal cancer

**DOI:** 10.1038/s41598-021-84475-4

**Published:** 2021-03-03

**Authors:** Takehito Yamamoto, Kenji Kawada, Koya Hida, Ryo Matsusue, Yoshiro Itatani, Rei Mizuno, Takashi Yamaguchi, Iwao Ikai, Yoshiharu Sakai

**Affiliations:** 1 Department of Surgery, Graduate School of Medicine, Kyoto University, 54 Shogoin-Kawara-cho, Sakyo-ku, Kyoto, 606-8507 Japan; 2 Department of Surgery, National Hospital Organization, Kyoto Medical Center, Kyoto, Japan

**Keywords:** Cancer, Gastroenterology, Oncology

## Abstract

Although numerous studies have highlighted the prognostic values of various inflammation-related markers, clinical significance remains to be elucidated. The prognostic values of inflammation-related biomarkers for rectal cancer were investigated in this study. A total of 448 patients with stage II/III rectal cancer undergoing curative resection were enrolled from the discovery cohort (n = 240) and validation cohort (n = 208). We comprehensively compared the prognostic values of 11 inflammation-related markers-derived from neutrophil, lymphocyte, platelet, monocyte, albumin, and C-reactive protein for overall survival (OS) and recurrence-free survival (RFS). Among 11 inflammation-related markers, only “lymphocyte × albumin (LA)” was significantly associated with both OS and RFS in the discovery cohort (*P* = 0.007 and 0.015, respectively). Multivariate analysis indicated that low LA was significantly associated with poor OS (hazard ratio [HR] 2.19, 95% confidence interval [CI] 1.09–4.58, *P* = 0.025), and poor RFS (HR 1.61, 95% CI 1.01–2.80, *P* = 0.048). Furthermore, using the discovery cohort, we confirmed that low LA was significantly associated with poor OS (HR 2.89, 95% CI 1.42–6.00, *P* = 0.002), and poor RFS (HR 1.79, 95% CI 1.04–2.95, *P* = 0.034). LA can be a novel prognostic biomarker for stage II/III rectal cancer.

## Introduction

Colorectal cancer (CRC) is one of the main causes of cancer death in the world. Despite recent advancements in surgical procedures and anti-cancer drugs, the mortality and recurrence of CRC remain high. In particular, rectal cancer patients exhibit poorer prognosis than colon cancer patients mainly due to the higher recurrence rate, and the treatment strategy of rectal cancer is different from that of colon cancer^1–3^. Identification of a subpopulation with a high risk of recurrence is clinically important for the treatment of rectal cancer in order to implement multidisciplinary approaches including extended surgery, preoperative treatment, adjuvant chemotherapy, and close follow-up. Therefore, an optimal prognostic biomarker of rectal cancer is needed in clinical practice.

Systemic Inflammation via tumor-stromal interaction is generally considered to be intensely involved in tumor progression^4–6^. Accumulating studies have indicated that several inflammation-related markers based on circulating blood cell counts may be useful prognostic factors in various malignancies^7–19^: e.g., neutrophil-to-lymphocyte ratio (NLR), lymphocyte-to-monocyte ratio (LMR), platelet-to-lymphocyte ratio (PLR), Glasgow prognostic score (GPS), lymphocyte-to-C-reactive protein (CRP) ratio (LCR), and systemic inflammation score (SIS). We have recently reported the combination of GPS and NLR can be useful to predict the prognosis of CRC patients^14^. Although many previous reports indicated the prognostic impact of circulating blood cell-based markers, the best combination of inflammation-related factors for predicting patients’ prognoses remains to be elucidated. Because the status of tumor microenvironment can be different depending on the cancer types, it is mandatory to focus specifically on the individual cancer in order to identify the optimal inflammation-related marker.

In the present study, we comprehensively compared the prognostic potentials of various combinations of circulating blood cell-based markers in the discovery cohort of patients with stage II/III rectal cancer who underwent curative resection. We also investigated the prognostic factors that affected overall survival (OS) and recurrence-free survival (RFS) by univariate and multivariate analyses. In this cohort, we found that “lymphocyte × albumin (LA)”, the product of the lymphocyte count multiplied by the albumin concentration, was particularly associated with rectal cancer patients’ prognosis; thus, we further explored the potential feasibility of the newly developed factor, “LA”, as a prognostic marker in the external validation cohort.

## Results

### Patient characteristics of the discovery cohort

Clinicopathological characteristics are indicated in Table 1. A total of 240 patients comprised 163 males and 77 females, and their median age was 66 years old [range 21–90] were included. Tumor location was Ra in 122 patients (51%), and Rb–P in 118 patients (49%). As a preoperative treatment, NAC was administered in 46 patients (19%) and nCRT in 24 patients (10%). The 5-year OS and RFS of all the 240 patients were 87.4% and 72.6%, respectively.

**Table 1:** Clinicopathological characteristics of study patients.

Variables	n = 240, median [range], n(%)
**Age**	66 [21–90]
**Sex**	
Male	163 (68)
Female	77 (32)
**Tumor location**	
Ra	122 (51)
Rb-P	118 (49)
**cStage**	
II	73 (30)
III	167 (70)
CEA	16.4 ± 9.7
**Preoperative treatment**	
none	170 (71)
nCRT	24 (10)
NAC	46 (19)
**Surgical procedure**	
LAR	158 (66)
ISR	32 (13)
APR	39 (16)
TPE	4 (2)
Hartmann	7 (3)
**Surgical approach**	
Laparoscopic	201 (84)
Robot	23 (10)
Open	10 (4)
Conversion	6 (2)
**pT classification**	
pT1/T2	75 (31)
pT3/T4	165 (69)
**pN classification**	
negative	157 (65)
positive	83 (35)
**Histology**	
well/mod	223 (93)
por/muc	16 (7)
**Adjuvant Chemotherapy**	107 (45)
**Inflammation parameters**	
Neutrophil (/µL)	3467 [768–13,275]
Lymphocyte (/µL)	1472 [67.2–28,079]
Platelet (/µL)	224,500 [16,000–523,000]
Monocyte (/µL)	341 [151–1344]
Albumin (g/dL)	4.0 [2.0–4.9]
CRP (mg/dL)	
0	57 (24)
0 < , ≤ 0.2	124 (52)
0.2 < , ≤ 1.0	45 (19)
1.0 <	14 (5)
**Combinations**	
NLR	2.31 [0.15–64.4]
PLR	140 [10–286]
PAR	56,300 [4000–223,000]
LMR	4.53 [0.24–81.06]
MAR	85.2 [32.7–413.3]
NP	767,460 [20,748–5,408,480] (× 10^3^)
LA	5950 [275.5–10,951]
NM	1,219,048 [227,697–13,069,600]
MP	76,682 [3057–322,560] (× 10^3^)
**GPS**	
0	224 (93)
1	6 (3)
2	10 (4)
**SIS**	
0	66 (28)
1	115 (48)
2	59 (24)

*CEA* carcinoembryonic antigen, *nCRT* neoadjuvant chemoradiotherapy, *NAC* neoadjuvant chemotherapy, *LAR* low anterior resection, *ISR* intersphincteric resection, *APR* abdominoperineal resection, *TPE* total pelvic exenteration, *well/mod* well-differentiated/moderately-differentiated, *por/muc* poorly-differentiated/mucinous, *CRP* C-reactive protein, *NLR* neutrophil-to-lymphocyte ratio, *PLR* platelet-to-lymphocyte ratio, *PAR* platelet-to-albumin ratio, *LMR* lymphocyte-to-monocyte ratio, *MAR* monocyte-to-albumin ratio, *NP* neutrophil × platelet, LA lymphocyte × albumin, *NM* neutrophil × monocyte, and *MP* monocyte × platelet, *GPS* Glasgow prognostic scale, *SIS* systemic inflammation scale.

### Prognostic impact of inflammation-related markers in the discovery cohort

**Figure 1:**
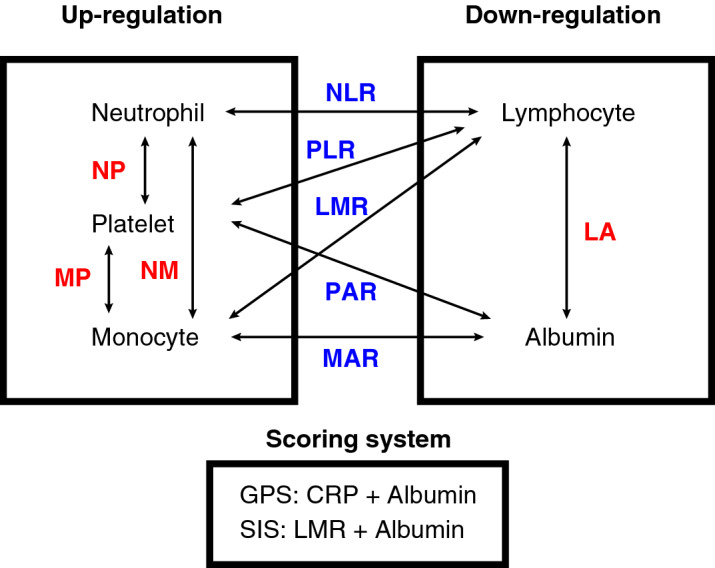
A schema of the 11 inflammation-related markers adopted in the present study. As systemic inflammatory factors, we chose 5 parameters (neutrophil, lymphocyte, platelet, monocyte, and albumin). The combination of each parameter (i.e., a total of 9 possible combinations) as well as two established inflammation-related scoring systems (i.e., GPS and SIS) were investigated.

As inflammation-related key factors, we adopted 5 parameters; up-regulation variables in disease progression were three parameters (neutrophil, platelet, and monocyte), whereas down-regulation variables in disease progression were two parameters (lymphocyte and albumin)^15,16,20,21^. To evaluate their prognostic impact in rectal cancer, the combination of each parameter (i.e., a total of 9 possible combinations), as well as two established inflammation-related scoring systems (i.e., GPS and SIS), were investigated (Fig. 1). These 11 inflammation-related markers (i.e., 9 combinations and 2 scoring systems) employed in this study are indicated in Table 1. Regarding GPS, the patients were categorized as GPS = 0 (n = 224; 93%), 1 (n = 6; 3%), and 2 (n = 10; 4%). Regarding SIS, the patients were categorized as SIS = 0 (n = 66; 28%), 1 (n = 115; 48%), and 2 (n = 59; 24%). Supplementary Fig. S1 shows distribution diagrams of 6 key factors (i.e., neutrophil, lymphocyte, platelet, monocyte, albumin, and CRP). Neutrophil count, monocyte count, platelet count, lymphocyte count, and albumin concentration followed normal distributions, whereas CRP level showed a skewed distribution. Among 240 patients, 181 patients (75%) had CRP level ≤ 0.2 mg/dL.

The prognostic impacts of the 11 inflammation-related markers were investigated by the log-rank test with the Kaplan–Meier method (Table 2). Univariate analysis indicated that low LA, the product of the lymphocyte count multiplied by the albumin concentration, was significantly associated with both poor OS and RFS (*P* = 0.007 and 0.015, respectively). The remaining combinations other than LA were not correlated with either OS or RFS in this cohort. Similarly, the 2 scoring systems (i.e., GPS and SIS) were not correlated with OS or RFS (Supplementary Fig. S2).

**Table 2:** The relationships between inflammation-related markers (the 9 combinations and 2 scoring systems) and survival.

Variables	OS		RFS	
	5-year OS (%)	*P* value	5-year RFS (%)	*P* value
**NLR**				
> 2.3	87.4		71.2	
≤ 2.3	87.4	0.165	74.0	0.293
**PLR**				
> 140	84.9		66.6	
≤ 140	89.6	0.069	78.9	0.088
**PAR**				
> 56,300	89.9		74.6	
≤ 56,300	85.1	0.847	70.8	0.900
**LMR**				
> 4.53	87.2		73.8	
≤ 4.53	87.7	0.462	71.2	0.517
**MAR**				
> 85.2	91.6		79.2	
≤ 85.2	83.6	0.572	66.5	0.111
**NP**				
> 767,000 (× 10^3^)	89.7		77.3	
≤ 767,000 (× 10^3^)	85.2	0.841	68.0	0.311
**LA**				
> 5950	91.1		80.4	
≤ 5950	83.1	0.007*	63.8	0.015*
**NM**				
> 1,210,000	90.1		78.5	
≤ 1,210,000	84.7	0.610	66.5	0.212
**MP**				
> 76,600 (× 10^3^)	91.2		78.0	
≤ 76,600 (x 10(× 10^3^)	84.1	0.254	67.9	0.281
**GPS**				
0	86.5		71.7	
1	100.0		83.3	
2	100.0	0.526	88.8	0.494
**SIS**				
0	89.1		79.2	
1	84.9		67.6	
2	91.1	0.499	74.3	0.226

*NLR* neutrophil-to-lymphocyte ratio, *PLR* platelet-to-lymphocyte ratio, *PAR* platelet-to-albumin ratio, *LMR* lymphocyte-to-monocyte ratio, *MAR* monocyte-to-albumin ratio, *NP* neutrophil × platelet, *LA* lymphocyte × albumin, *NM* neutrophil × monocyte, *MP* monocyte × platelet, *GPS* Glasgow prognostic scale, *SIS* systemic inflammation scale.

* *P* < 0.05.

During the study period, there were 37 deaths (15%) and 55 recurrences (23%). We further analyzed receiver-operating characteristic curves of the 9 combinations, and found that LA had the highest area under the curve values to predict OS and RFS (0.58 and 0.58, respectively) (Supplementary Figs. S3, S4).

**Figure 2:**
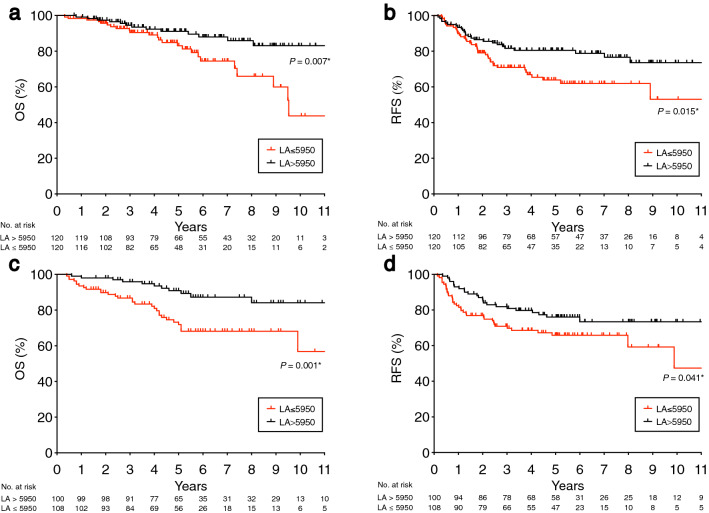
Prognostic impact of LA on OS and RFS in the discovery cohort (**a**,**b**) and validation cohort (**c**,**d**). Cox’s proportional hazard regression model. (**a**,**c**) LA > 5950 or ≤ 5950 for OS, (**b**,**d**) LA > 5950 or ≤ 5950 for RFS.

Next, we investigated potential prognostic factors for OS (Table 3, left). Univariate analysis showed that poor OS was significantly associated with older age (> 65 years), high CEA level (> 5 ng/mL), pN category (pN+), histology (por/muc), and low LA (≤ 5950). On multivariate analysis using Cox’s proportional hazard regression model, older age (hazard ratio [HR] 2.55, 95% confidence interval [CI] 1.21–5.74, *P* = 0.012), pN+ (HR 2.06, 95% CI 1.02–4.15, *P* = 0.042) and low LA (HR 2.19, 95% CI 1.09–4.58, *P* = 0.025) remained to be significantly associated with poor OS. Figure 2a shows the Kaplan–Meier curve of LA on OS.

**Table 3:** Univariate and multivariate analysis using clinicopathological characteristics and LA for overall survival (OS) and recurrence-free survival (RFS) in the discovery cohort.

Variables	OS						RFS				
	Univariate			Multivariate			Univariate		Multivariate		
	n	5-year OS (%)	*P* value	HR	95%CI	*P* value	5-year RFS (%)	*P* value	HR	95%CI	*P* value
**Age**											
> 65	138	82.5					69.6				
≤ 65	102	93.6	< 0.001*	2.55	1.21–5.74	0.012*	75.7	0.254			
**Sex**											
Male	163	86.9					72,1				
Female	77	88.3	0.927				73.7	0.948			
**Tumor location**											
Ra	122	86.7					76.9				
Rb-P	118	87.7	0.198				68.5	0.352			
**CEA**											
> 5	99	83.5					66.4				
≤ 5	141	90.0	0.008*	1.75	0.85–3.72	0.125	76.6	0.071	1.13	0.67–1.89	0.638
**pT category**											
pT1/2	75	93.4					87.9				
pT3/4	165	84.7	0.051	1.39	0.58–3.87	0.473	65.5	0.001*	2.30	1.19–4.90	0.011*
**pN category**											
pN−	157	91.2					81.0				
pN+	83	79.3	0.042*	2.06	1.02–4.15	0.042*	55.8	< 0.001*	1.99	1.09–3.01	0.008*
**Neoadjuvant therapy**											
Present	70	90.7					70.8				
Absent	170	85.9	0.465				73.4	0.968			
**Histology**											
Well/mod	223	88.7					74.6				
Por/muc	16	70.7	0.006*	1.81	0.62–4.55	0.257	43.7	0.001*	2.21	1.00–4.08	0.049*
**Adjuvant chemotherapy**											
Present	107	90.5					71.7				
Absent	133	84.7	0.055	1.88	0.92–4.10	0.086	73.5	0.522			
**LA**											
> 5950	120	91.1					80.4				
≤ 5950	120	83.1	0.007*	2.19	1.09–4.58	0.025*	63.8	0.015*	1.61	1.01–2.80	0.048*

*CEA* carcinoembryonic antigen, *well/mod* well-differentiated/moderately-differentiated, *por/muc* poorly-differentiated/mucinous, *LA* lymphocyte × albumin, *HR* hazard ratio, *CI* confidence interval.

* *P* < 0.05.

We also investigated potential prognostic factors for RFS (Table 3, right). Univariate analysis showed that poor RFS was significantly associated with pT category (pT3/4), pN category (pN+), histology (por/muc), and low LA (≤ 5950). On multivariate analysis using Cox’s proportional hazard regression model, pT3/4 (HR 2.30, 95% CI 1.19–4.90, *P* = 0.011), pN+ (HR 1.99, 95% CI 1.09–3.01, *P* = 0.008), histology (por/muc) (HR 2.21, 95% CI 1.00–4.08, *P* = 0.049), and low LA (HR 1.61, 95% CI 1.01–2.80, *P* = 0.048) remained to be significantly associated with poor RFS. Figure 2b shows the Kaplan–Meier curve of LA on RFS. * *P* < 0.05

### Prognostic value of LA in the validation cohort

Next, we investigated the prognostic potential of LA in the external validation cohort (n = 208). Clinicopathological characteristics of the validation cohort are presented in Supplementary Table S1. Univariate and multivariate analyses for OS were conducted using clinicopathological characteristics and LA (Table 4, left). Of note, multivariate analysis using Cox’s proportional hazard regression model indicated that low LA was significantly associated with poor OS (HR 2.89, 95% CI 1.42–6.00, *P* = 0.002). Figure 2c shows the Kaplan–Meier curve of LA.

**Table 4:** Univariate and multivariate analysis using clinicopathological characteristics and LA for overall survival (OS) and recurrence-free survival (RFS) in the validation cohort.

Variables	OS						RFS				
	Univariate			Multivariate			Univariate		Multivariate		
	n	5-year OS (%)	*P* value	HR	95%CI^f^	*P* value	5-year RFS (%)	*P* value	HR	95%CI	*P* value
**Age**											
> 65	121	80.4					75.3				
≤ 65	87	83.2	0.598				64.6	0.147			
**Sex**											
Male	130	79.9					66.4				
Female	78	84.2	0.481				78.0	0.102			
**Tumor location**											
Ra	101	82.7					71.6				
Rb-P	107	80.3	0.923				69.8	0.712			
**CEA**											
> 5	99	75.6					65.3				
≤ 5	107	87.3	0.019*	1.44	0.74–2.84	0.275	75.8	0.114			
**pT category**											
pT1/2	39	93.9					83.6				
pT3/4	169	78.4	0.063	2.33	0.89–8.01	0.085	67.8	0.026*	2.81	1.30–7.35	0.006*
**pN category**											
pN−	98	89.6					84.1				
pN+	110	74.4	0.002*	3.28	1.59–7.22	< 0.001*	58.8	< 0.001*	3.59	2.05–6.66	< 0.001*
**Neoadjuvant therapy**											
Present	32	69.1					52.1				
Absent	176	83.8	0.019	2.82	1.38–5.87	0.006*	74.2	0.014*	2.30	1.23–4.06	0.009*
**Histology**											
Well/mod	200	81.5					70.2				
Por/muc	6	66.6	0.969				75.0	0.848			
**Adjuvant** **chemotherapy**											
Present	73	83.8					70.3				
Absent	135	81.1	0.393				70.9	0.816			
**LA**											
> 5950	100	90.8					76.0				
≤ 5950	108	73.5	0.001*	2.89	1.42–6.00	0.002*	65.8	0.041*	1.79	1.04–2.95	0.034*

*CEA* carcinoembryonic antigen, *well/mod* well-differentiated/moderately-differentiated, *por/muc* poorly-differentiated/mucinous, *LA* lymphocyte × albumin, *HR* hazard ratio, *CI* confidence interval.

* *P* < 0.05

Furthermore, univariate and multivariate analyses for RFS were conducted using clinicopathological characteristics and LA (Table 4, right). Multivariate analysis using Cox’s proportional hazard regression model indicated that low LA remained to be significantly associated with poor RFS (HR 1.79, 95% CI 1.04–2.95, *P* = 0.034). Figure 2d shows the Kaplan–Meier curve of LA. Collectively, these results regarding the prognostic potential of LA in the validation cohort were almost consistent with those in the discovery cohort.

### Stratification of the prognostic impact of LA levels

**Figure 3:**
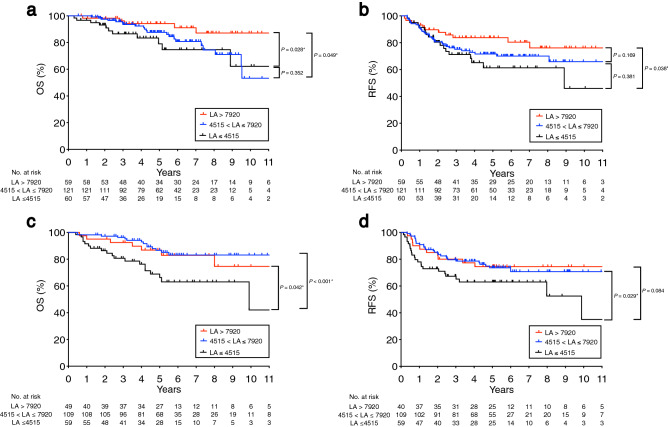
Stratification of the prognostic impact of LA levels in the discovery cohort (**a**,**b**) and validation cohort (**c**,**d**). Comparison of prognostic impact on OS (**a**,**c**) and RFS (**b**,**d**) among three groups; higher LA group (LA > 7920), middle LA group (4515 < LA ≤ 7920), and lower LA group (LA ≤ 4515). The upper quartile and the lower quartile of LA were 7920 and 4515, respectively. * *P* < 0.05

These aforementioned results indicated that LA was a robust biomarker for rectal cancer patients’ prognosis. Therefore, we further investigated the prognostic value among the different LA levels. In the discovery cohort, the upper and lower quartiles of LA were 7920 and 4515, respectively. All 240 patients were classified into three groups based on the interquartile ranges of LA: (1) higher LA group (LA > 7920; n = 59), (2) middle LA group (4515 < LA ≤ 7920; n = 121), and (3) lower LA group (LA ≤ 4515; n = 60). Kaplan–Meier curves of the three groups on OS and RFS are shown in Fig. 3a,b. The lower LA group exhibited much poorer prognosis on OS and RFS than the higher LA group (*P* = 0.028 and *P* = 0.038, respectively).

Furthermore, we perform the same analysis in the validation cohort. All 208 patients were classified into three groups using the same cut-off values: (1) higher LA group (LA > 7920; n = 40), (2) middle LA group (4515 < LA ≤ 7920; n = 109), and (3) lower LA group (LA ≤ 4515; n = 59). Kaplan–Meier curves of the three groups on OS and RFS are shown in Fig. 3c,d. The lower LA group exhibited much poorer prognosis on OS and RFS than the higher LA group (*P* = 0.042 and *P* = 0.084, respectively) and middle LA group (*P* < 0.001 and *P* = 0.029, respectively). Taken together, these results indicated that LA could be a useful marker to predict the prognosis of rectal cancer.

## Discussion

Although a growing number of studies have highlighted the prognostic values of various inflammation-related markers in several types of cancer^7–19^, clinical significance of inflammation-related markers remains to be elucidated. Moreover, there is a lack of consensus about the cut-off values for each marker. In this study, we focused on the patients with stage II/III rectal cancer, because accurate prediction of their prognosis can help decision-making in postoperative management, such as the follow-up schedule and need for adjuvant chemotherapy. In addition, the treatment strategy for stage IV rectal cancer is more complex compared with that for stage II or III, which indicates the need to investigate stage IV rectal cancer separately.

In the present study, we comprehensively evaluated the predictive potential for prognosis of 11 inflammation-related biomarkers (i.e., NLR, PLR, LMR, PAR, MAR, NP, LA, NM, MP, GPS, and SIS) to identify the best combination of peripheral blood cell-based markers for stage II/III rectal cancer, and found that LA was a more reliable prognostic predictor compared with any other inflammation-related markers in the discovery cohort (Tables 2, 3; Fig. 2a,b; Supplementary Fig. S3, S4). Of note, this prognostic impact of LA could be almost confirmed using the external validation cohort (Table 4; Fig. 2c,d). Although the potential causal effect behind the association of the LA with prognosis is not clear, it is possible to postulate the following hypotheses. Lymphocytes play a crucial role in the host cell-mediated cytotoxic immune response against tumors. A high density of tumor-infiltrating lymphocytes is strongly correlated with a favorable prognosis in several types of cancers^22^, suggesting that anti-tumor immune response is mediated mainly by lymphocytes. Serum albumin is produced from the liver, and is known as one of the negative acute phase proteins in response to inflammation. Furthermore, low albumin concentration also means malnutrition, and this can negatively affect tumor immunity in the microenvironment. Considering these findings, LA may reflect both the immunological response, represented by the lymphocyte count, and nutritional status, represented by the serum albumin level.

Serum CRP level is a well-known established inflammatory marker. Combinations including the preoperative CRP level have been reported as prognostic markers in CRC. A recent study reported a significant potential of LCR, lymphocyte-CRP ratio. Okugawa et al. compared 9 inflammation-related markers in a discovery cohort of 373 patients with stage I–IV CRC, and found that LCR was a new reliable prognostic marker compared with other markers^15^. Suzuki et al. also reported LCR was an independent predictive marker for OS and RFS in 1303 patients with stage II/III CRC^16^. In our institution, however, CRP levels lower than 0.1 mg/dL were judged as “zero”; thus, they could not be multiplied or divided by another variable. Furthermore, the distribution of serum CRP levels was too skewed (Table 1; Supplementary Fig. S1). Among 240 patients, 181 patients (75%) had CRP levels ≤ 0.2 mg/dL, that is, most of the study patients had the CRP levels within the normal range. Therefore, we assume that the variables incorporating the preoperative CRP level are difficult to become candidates for prognostic markers. Meanwhile, it is worth noting that the distribution of LA followed a normal distribution.

Although we have recently reported the combination of GPS and NLR can be useful to predict the prognosis of CRC patients^14^, we could not identify the prognostic value of either NLR or GPS in this study (Table 2; Supplementary Fig. S2a,b). Recently, Suzuki et al. reported that SIS was a powerful predictor of OS and RFS in 727 patients with Stage I–IV CRC^17^. However, we could not identify the prognostic value of SIS in the discovery cohort (Table 2, Supplementary Fig. S2c,d). These differences might be due to our focusing on rectal cancer or our exclusion of stage I and IV cancers.

This study has some limitations. First, this was a retrospective study with a relatively small population, and thus may be influenced by selection bias. Second, our treatment strategy was subject to certain chronological changes. Third, we included the patients with short observational period or censored cases shortly after operation, and this is a limitation to construct a prediction model for prognosis. In the future, we need to reevaluate the prediction performance of LA using ROC analysis or a deep learning method after a long period of observation^23,24^. Thus, further studies are desirable to establish the clinical utility of the newly developed LA.

As far as we know, this is the first report to reveal the prognostic value of LA in rectal cancer. In stage II/III rectal cancer, assessment of preoperative LA can identify the high-risk subgroup for recurrence, which might help physicians to decide the postoperative management for preventing recurrence.

In conclusion, we found that “lymphocyte × albumin (LA)”, the product of the lymphocyte count multiplied by the albumin concentration was a novel prognostic biomarker for stage II/III rectal cancer patients. This novel marker is easily calculated through routine blood tests, which can provide opportunities for further investigation.

## Materials and methods

### Patient population

A total of 448 patients with clinical Stage II/III rectal cancer undergoing curative operation were enrolled from two different cohorts: 240 patients from Kyoto University Hospital between 2005 and 2017 as the discovery cohort, and 208 patients from National Hospital Organization Kyoto Medical Center between 2006 and 2017 as the validation cohort. Median observational period in these cohorts were 58 [3.6–152] months and 60 [4.8–156] months, respectively.

In all cases, the tumor was within 10 cm from the anal verge. Tumor location was defined according to the Japanese classification of Colorectal Carcinoma: “Ra”, was the portion between S2 sacral vertebra and peritoneal reflection, “Rb” was the portion between peritoneal reflection and anal canal, and “P” was the portion within the anal canal^25^. Clinical stage was determined based on colonoscopy, magnetic resonance imaging (MRI), and computed tomography (CT). The institutional review board of Kyoto University approved the study protocol (reference No. R1958), and conformed to the Declaration of Helsinki. Informed consent was obtained from all participants.

### Treatment protocol

All surgeries were conducted by board-certified laparoscopic colorectal surgeons^26^. Treatment strategies of individual cases were determined based on the multidisciplinary team meeting. Preoperative treatment (neoadjuvant chemoradiotherapy [nCRT] or neoadjuvant chemotherapy) was administered for patients with a high risk of recurrence, such as marked enlargement of regional lymph nodes, or bulky tumors (e.g., tumor size > approximately 50 mm, or clinically suspected involvement of circumferential resection margin)^27^. In the nCRT group, radiation therapy (total 45 Gy in 25 fractions) with concomitant CPT-11 and S-1 was administered. In the NAC group, FOLFIRI-based chemotherapy, FOLFOX6-based chemotherapy, or CPT-11 plus S-1 regimen was administered.

### Follow-up investigation

The patient follow-up was standardized as follows; physical examination and blood test were done every 3 months in the first 3 years and every 6 months thereafter. CT was done every 6 months (for stage II) or every 3 months (for stage III) in the first 3 years, and subsequently continued every 6 months for more than 2 years. Colonoscopy was done at 1, 3, and 5 years after operation. Recurrence was diagnosed by the imaging examinations and histopathological findings. OS was measured from the date of the first surgery to the date of death. RFS was measured from the date of the first surgery to the date of cancer recurrence or the date of death.

### Inflammation-related markers

Blood samples were collected within one month before the surgery. In order to identify the inflammation-related prognostic markers, we chose 5 parameters available in a routine blood test: neutrophil, lymphocyte, platelet, monocyte, and albumin (Fig. 1). The 5 parameters were characterized as either up-regulating group (i.e., neutrophil, platelet, and monocyte) or down-regulating group (i.e., lymphocyte and albumin)^15,16,20,21^. Afterwards, we generated 9 combinations from the 5 parameters: NLR, PLR, LMR, LA, platelet-to-albumin ratio (PAR), monocyte-to-albumin ratio (MAR), neutrophil × platelet (NP), neutrophil × monocyte (NM), and monocyte × platelet (MP). When each of the combined parameters belonged to the same group, they were multiplied; when they belonged to different groups, they were divided. In addition, we chose GPS and SIS as the established inflammation-related scoring systems. Regarding GPS, patients with both higher CRP (> 1.0 mg/dL) and lower albumin concentration (< 3.5 g/dL) were categorized as GPS = 2, patients with either of these two were categorized as GPS = 1, and patients with neither were categorized as GPS = 0^11,19^. Regarding SIS, patients with both lower albumin concentration (< 4.0 g/dL) and lower LMR (< 4.44) were categorized as SIS = 2, patients with either of these two were categorized as SIS = 1, and patients with neither were categorized as SIS = 0^17,18^. These 11 inflammation-related markers composed of the 9 combinations and 2 scoring systems (GPS and SIS) were investigated to identify the prognostic biomarker with the highest accuracy in rectal cancer (Fig. 1).

### Prognostic analysis

First, the prognostic impact of the 11 inflammation-related markers on OS and RFS were analyzed in the discovery cohort (n = 240). The cut-off values of the 9 combinations were determined referring to the respective median values. Next, the potential markers identified as statistically significant, as well as clinicopathological variables [i.e., age, sex, tumor location, carcinoembryonic antigen (CEA), pT category, pN category, neoadjuvant therapy, and histology], were analyzed by multivariate analysis. Finally, the results obtained from the discovery cohort were evaluated in the external validation cohort (n = 208).

### Statistical analysis

Continuous variables are presented as mean ± standard deviation (SD) or median. Survival outcomes were analyzed by the log-rank test with the Kaplan–Meier method. Variables with a *P* value less than 0.1 in the univariate analysis were included in the multivariate analysis using the Cox’s proportional hazard regression model. All analyses were two-sided, and a *P* value less than 0.05 was defined statistically significant. All statistical analyses were conducted using JMP Pro, Version 14 (SAS Institute Inc., Cary, NC, USA).

## Supplementary Information

Supplementary Information
