# Supplementary material for: Combination of lymphocyte count and albumin concentration as a new prognostic biomarker for rectal cancer
Source: Sci Rep. 2021 Mar 3;11:5027. doi: 10.1038/s41598-021-84475-4 (PMC7930240; doi:10.1038/s41598-021-84475-4)

Supplementary Table S1. Clinicopathological characteristics of the validation cohort

Variables	Validation cohort (n=208)
Age	68 [31–103]
Sex	
Male	130 (63)
Female	78 (37)
Tumor location	
Ra	101 (49)
Rb-P	107 (51)
cCRM	
Positive	50 (24)
Negative	158 (76)
cT classification	
cT1/T2	37 (18)
cT3/T4	171 (82)
cStage	
II	105 (50)
III	103 (50)
CEA	16.4±40.9
Preoperative treatment	
None	176 (85)
nCRT	30 (14)
NAC	2 (1)
Histology	
Well/Moderate	200 (96)
Por/Muc	6 (4)

CEA carcinoembryonic antigen, nCRT neoadjuvant chemoradiotherapy, NAC neoadjuvant chemotherapy, LAR low anterior resection, ISR intersphincteric resection, APR abdominoperineal resection, TPE total pelvic exenteration, well/mod well-differentiated/moderately-differentiated, por/muc poorly-differentiated/mucinous

Supplementary Fig. S1. The distribution of 6 key factors (neutrophil, lymphocyte, platelet, monocyte, CRP, and albumin)

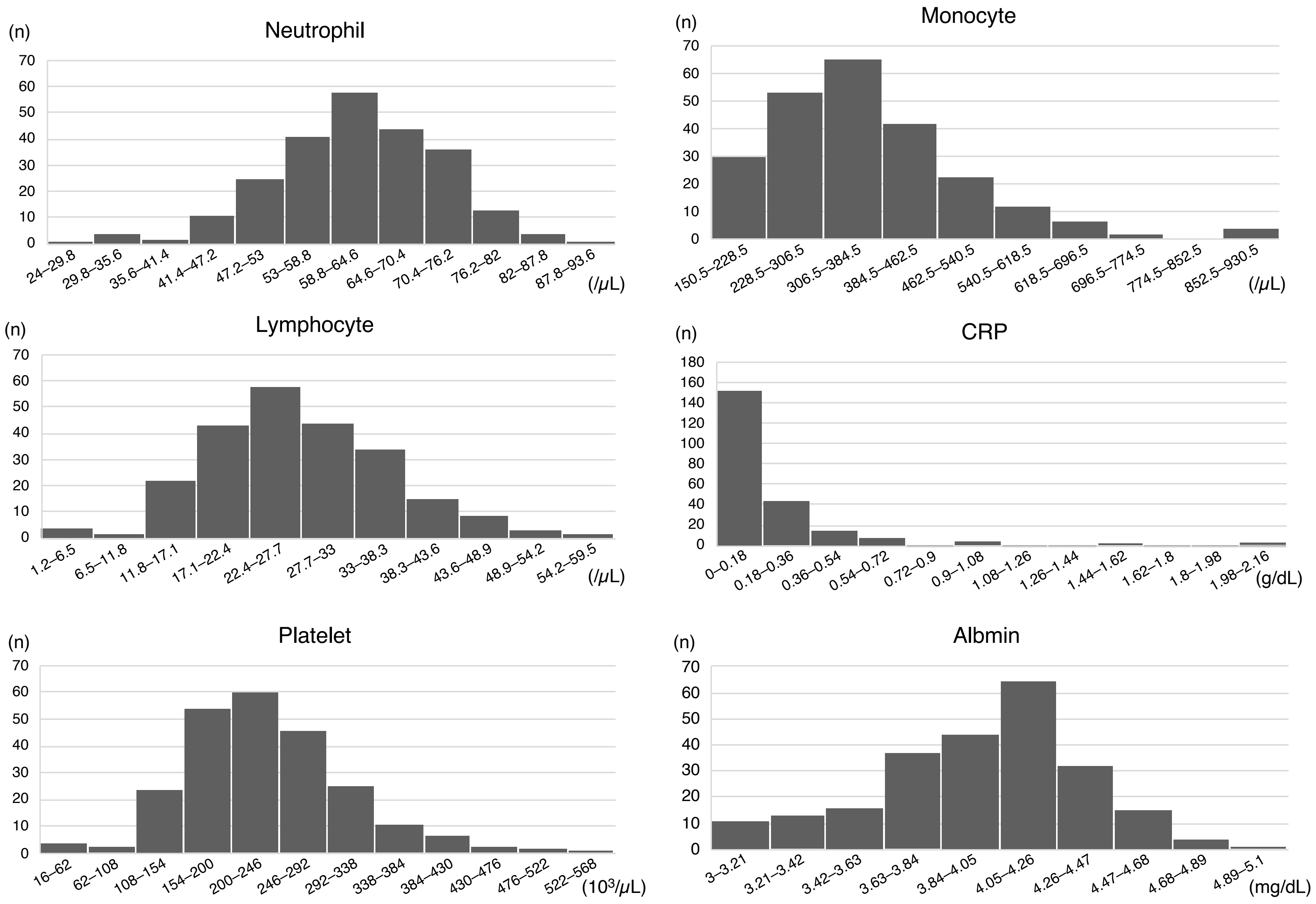

Supplementary Fig. S2. The prognostic impact of GPS and SIS for OS and RFS in Cox's proportional hazard regression model. **a** and **b**, GPS; **c** and **d**, SIS

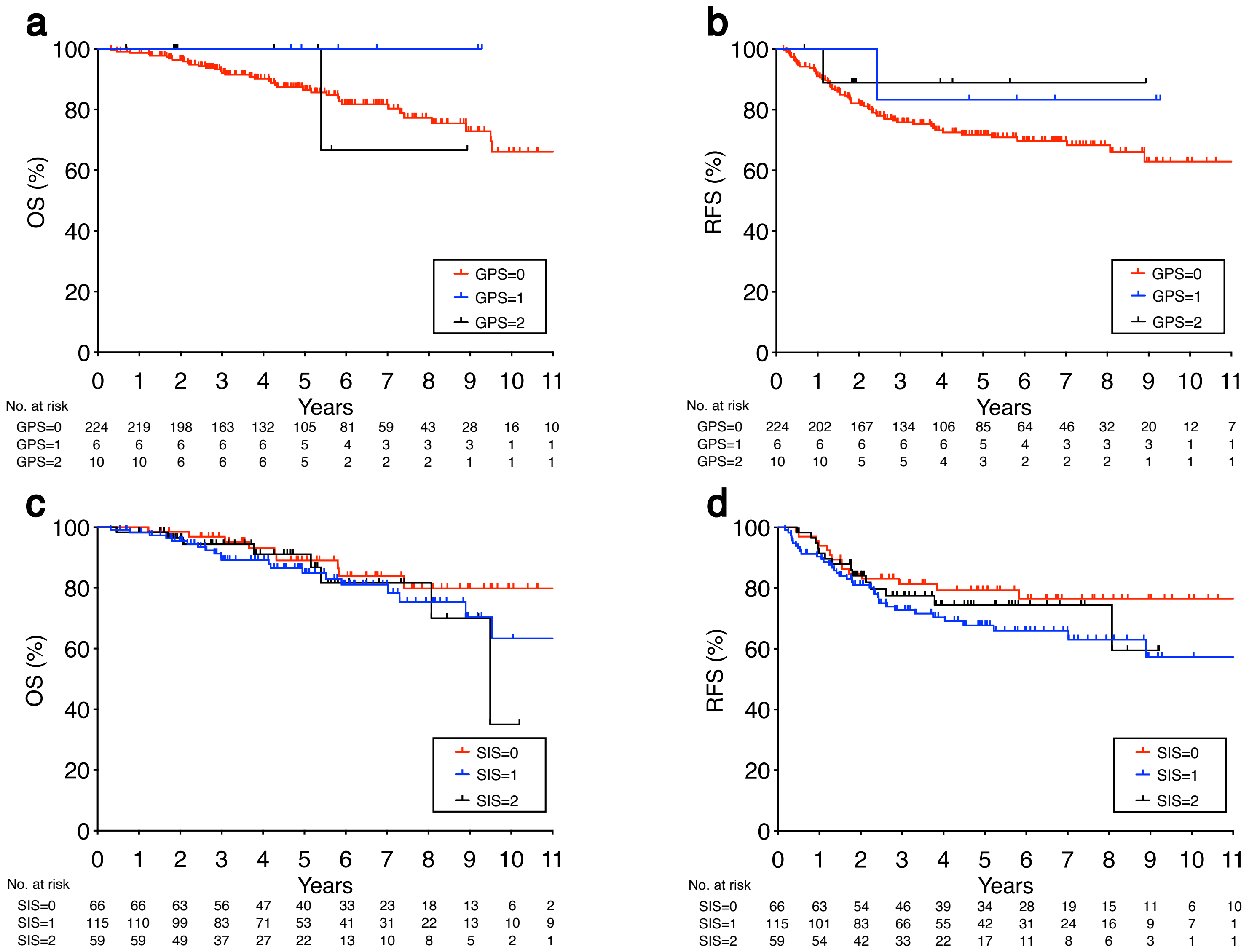

Supplementary Fig. S3. Receiver operating characteristics curve analysis to evaluate the predictive value of the 9 combinations for OS.

AUC: area under the curve

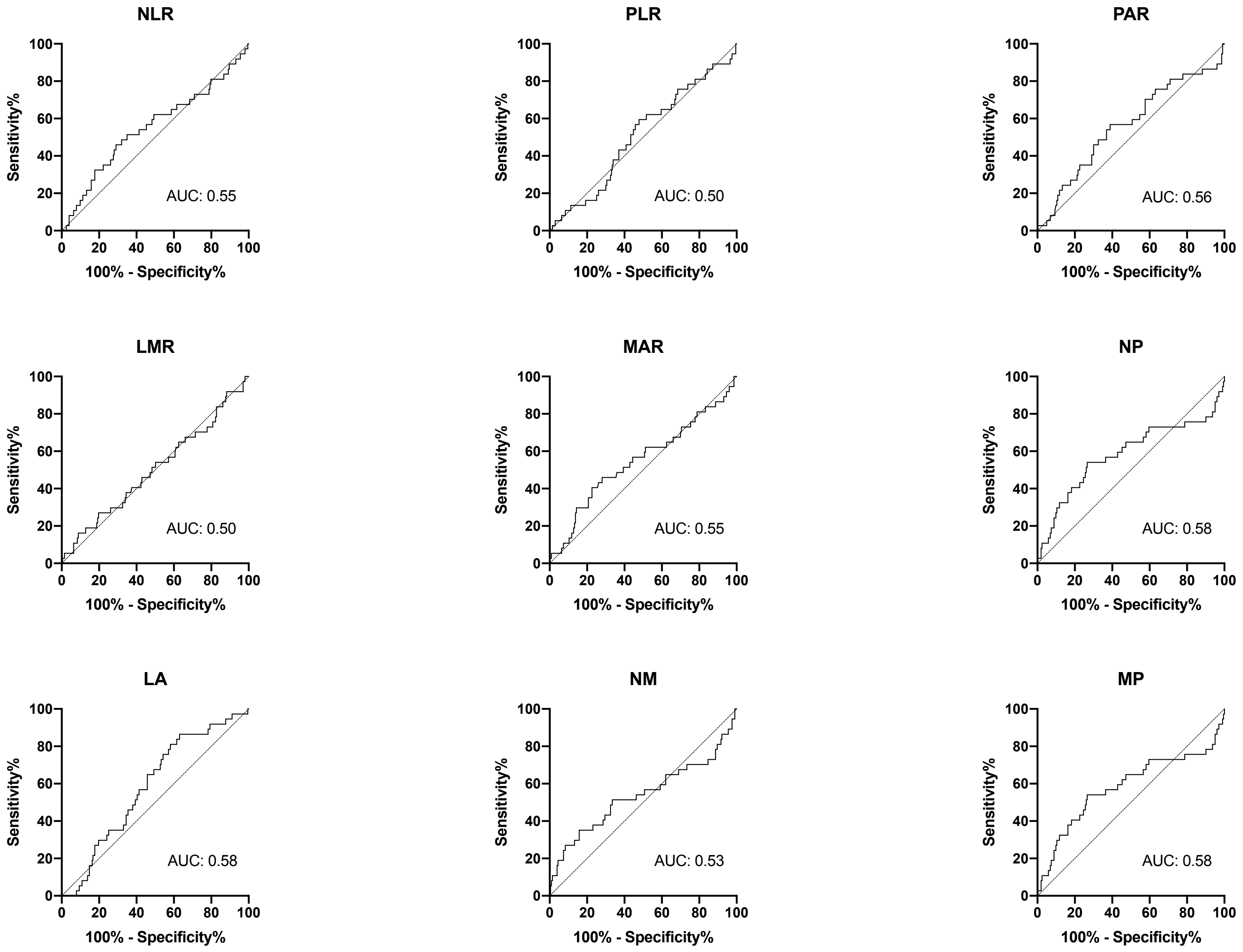

Supplementary Fig. S4. Receiver operating characteristics curve analysis to evaluate the predictive value of the 9 combinations for RFS.

AUC: area under the curve

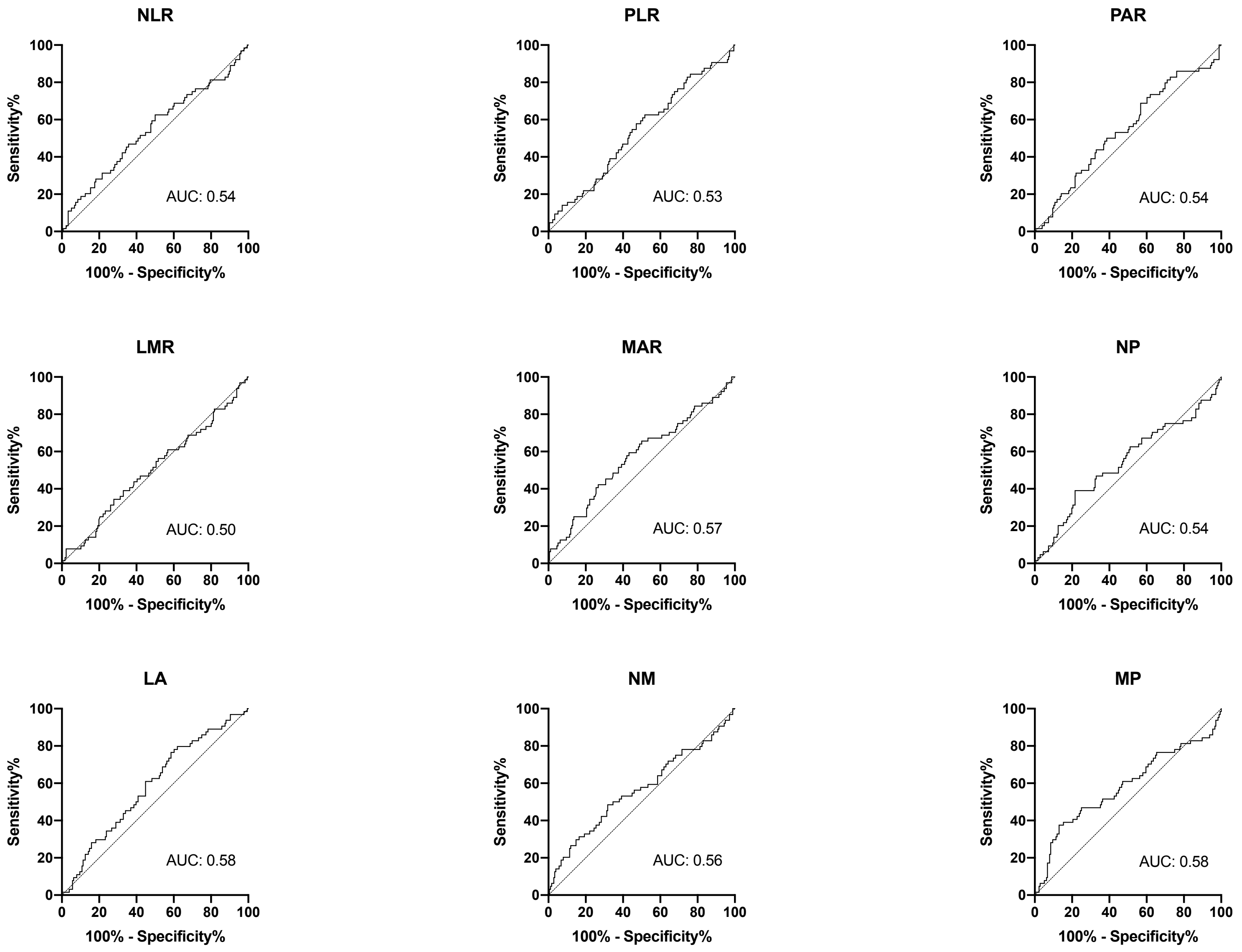

Supplement: Supplementary file 1 — Supplementary Information [file 41598_2021_84475_MOESM1_ESM.pdf]
